# Deep-Learning-Based Segmentation of Small Extracellular Vesicles in Transmission Electron Microscopy Images

**DOI:** 10.1038/s41598-019-49431-3

**Published:** 2019-09-13

**Authors:** Estibaliz Gómez-de-Mariscal, Martin Maška, Anna Kotrbová, Vendula Pospíchalová, Pavel Matula, Arrate Muñoz-Barrutia

**Affiliations:** 1 Bioengineering and Aerospace Engineering Department, Universidad Carlos III de Madrid, Leganés, 28911 Spain; 2 Instituto de Investigación Sanitaria Gregorio Marañón, Madrid, 28007 Spain; 3 Centre for Biomedical Image Analysis, Faculty of Informatics, Masaryk University, Brno, 602 00 Czech Republic; 4 Department of Experimental Biology, Faculty of Science, Masaryk University, Brno, 611 37 Czech Republic

**Keywords:** Extracellular signalling molecules, Image processing

## Abstract

Small extracellular vesicles (sEVs) are cell-derived vesicles of nanoscale size (~30–200 nm) that function as conveyors of information between cells, reflecting the cell of their origin and its physiological condition in their content. Valuable information on the shape and even on the composition of individual sEVs can be recorded using transmission electron microscopy (TEM). Unfortunately, sample preparation for TEM image acquisition is a complex procedure, which often leads to noisy images and renders automatic quantification of sEVs an extremely difficult task. We present a completely deep-learning-based pipeline for the segmentation of sEVs in TEM images. Our method applies a residual convolutional neural network to obtain fine masks and use the Radon transform for splitting clustered sEVs. Using three manually annotated datasets that cover a natural variability typical for sEV studies, we show that the proposed method outperforms two different state-of-the-art approaches in terms of detection and segmentation performance. Furthermore, the diameter and roundness of the segmented vesicles are estimated with an error of less than 10%, which supports the high potential of our method in biological applications.

## Introduction

Small extracellular vesicles (sEVs) are cell-derived nanoscale particles (~30–200 nm) involved in inter-cellular communication^1–3^. They are released by almost all cell types and transport biological information about the parental cell (proteins, lipids and microRNAs, among others)^4,5^. There is a fast growing interest in the characterizing of sEVs and in deciphering their role in cellular processes in both health and disease, with the hopes of bringing novel insights to the diagnoses and therapies for developing neurodegenerative diseases^6^, infections^7,8^, and cancer^9,10^.

Despite the existence of several EV subtypes, any consensus on their specific markers has not yet been reached. Assigning an EV to a particular biogenesis pathway upon its isolation from complex biological fluids is difficult. Therefore, in compliance with the 2018 guidelines^11^ released by the International Society for Extracellular Vesicles, we use a more generic term, sEVs, for nanoparticles until recently referred to as exosomes.

Since the last decade, thanks to new and more powerful characterization techniques in the nanoscale, it has been possible to study in depth the biophysical composition of sEVs and their role in cellular processes^12–14^. Specifically, sEV morphology studies are currently performed by a battery of techniques: Nanoparticle-tracking analysis (NTA)^15^, tunable-resistive pulse sensing (TRPS)^16^, flow cytometry (FC)^17^ and transmission electron microscopy (TEM) imaging^12,18^. All but TEM allow high-throughput, although accurate estimation of sEV size relies on the homogeneity of sEV populations and on the sphericity of sEV shapes. However, recent studies have derived evidence on the existence of distinguishable sEV groups^19^, namely due to the broad range of effects on recipient cells, which can only be explained if sEVs display heterogeneous characteristics. Recently, classifications of sEVs in sub-populations were proposed, being based on the biophysical characteristics and molecular compositions of sEVs^20^ or on the shape of sEVs^21^. Additionally, TEM provides real images of the vesicles formed by electron beams transmitted through a specimen instead of performing indirect measurements, which makes it a very suitable technique for the characterization of sEV morphology^18^.

Despite the benefits of TEM, there is a compromise between the information provided by electron microscopy (EM) images and the time required to extract it. As previously explained^22^, sample preparation for EM image acquisition has some risk of artifact generation, which interferes with the automation image processing. The common artifacts in TEM images include precipitated stain, protein aggregates and other impurities. Additionally, imperfect membranes on EM grids, inadequate staining of samples or anomalies created during acquisition of images also complicate automated EV analysis. Performing manual or semi-automatic measurements in EM images is extremely laborious and time-consuming. Moreover, the task is subjective and error-prone. The lack of fully automatic, accurate and fast methods for EM image processing impedes the routine use of quantitative analysis of sEVs in biomedical laboratories. It does also largely limit the throughput of the sample analysis.

Over the last few years, a myriad of techniques for the detection and segmentation of objects in different microscopy modalities have been reported^23,24^. Among others, Crescitelli *et al*.^20^ used the IMOD package^25^ to extract the size of sEVs from TEM images semi-automatically. Attempting to solve a similar task, multiple groups aimed to segment small compact objects such as mitochondria, vesicles or insulin granules from EM data^26–31^. Additionally, machine learning methods have achieved admirable results in EM image processing tasks^29,30,32–35^. Regarding cell detection and segmentation in optical microscopy images, convolutional neural networks (CNNs) have produced the most accurate results^23,24,36^. To the best of our knowledge, there is only one published method devoted to the automatic segmentation of sEVs in TEM images: TEM ExosomeAnalyzer^18,37^. It applies a pipeline of classical image processing routines to obtain a labeled mask of sEVs under the assumption that they are almost perfect spherical objects. TEM ExosomeAnalyzer requires manual curation of the detected sEV candidates to obtain biologically relevant measurements^18^.

In this work, we propose a robust, fully automatic deep-learning-based approach to segment sEVs in TEM images, allowing straightforward and automatic quantitative analyses of large datasets. We built our fully residual U-Net (FRU-Net) over a simplified version of the U-Net^38,39^ by adding residual layers^40^ to every convolutional layer. The FRU-Net provides fine masks of segmented sEVs that are post-processed for cluster splitting using the Radon transform^41^.

The output of the proposed approach is quantitatively evaluated and compared with the results of TEM ExosomeAnalyzer^18,37^ and with the U-Net itself^38,39^ to measure the effect of the modifications introduced in the FRU-Net architecture.

The main contributions of this work are therefore: (1) a robust TEM image processing method for sEV detection and segmentation; (2) a detailed comparison with state-of-the-art methods, (3) a demonstration of the biological relevance of the extracted morphological measurements (i.e., individual sEV diameter and roundness) and (4) the first publicly available set of manually annotated sEVs in TEM images.

## Materials and Image Data

The sEVs were isolated from cell culture media and ascites of ovarian cancer patients using differential centrifugation followed by a purification step in sucrose/D_2_O cushion [10.3402/jev.v4.25530]. Eight-microliter drops of sEVs in Phosphate Buffered Saline (PBS) were adsorbed by an activated formvar carbon coated EM grids (Pyser-SGI Limited) for 5–15 minutes at room temperature. Then, sEVs were stained with 2% ammonium molybdate for 20 seconds at room temperature. The samples were imaged using a Morgagni 268D (FEI) transmission electron microscope at different magnifications and at a voltage of 70 kV. Finally, the collected raw image data was exported as 16-bit grayscale TIFF images of size 2048 × 2048 pixels.

The images were split in three datasets to cover the substantial variability in sEV appearance in TEM images, see Supplementary Fig. S1. Dataset 1^37^ consisted of 20 heterogeneous images, containing 65 sEVs in total, imaged at random magnifications resulting in pixel sizes ranging from 0.26 nm to 0.63 nm. The images have a coarse and grainy background. Non-EV structures (artifacts) are present. Dataset 2 (an extended version of Dataset 1 in^18^) consisted of 14 homogeneous images, containing 346 sEVs in total, imaged at a fixed magnification resulting in a pizel size of 1.56 nm. Those images are cleaner, presenting a smooth background. In Dataset 3 (an extended version of Dataset 2 in^18^), part of the sEVs were isolated from culture media of several cell lines: Mouse fibroblasts (L-cells), mouse embryonic fibroblast (MEF), human embryonic kidney 293 (HEK-293), and the ovarian cancer cell line Kuramochi. Dataset 3 consisted of 38 highly heterogeneous images, containing 688 sEVs in total, imaged at different magnifications resulting in pixel sizes from 0.26 to 2.42 nm. Some of the images present a smooth background while the rest suffer from a coarse and grainy background. Non-EV structures (artifacts) are part of all the images in this dataset.

Each dataset presents a different level of complexity, Dataset 2 being the easiest to process and Dataset 3 the most challenging one. The whole set of images consists of 72 TEM images taken at various magnifications and 1.099 annotated sEVs of different sizes. The images were manually annotated by multiple human experts over several iterations until the experts reached the consensus Ground Truth. Typically, tens of minutes were spent on manual annotation of a single image with ~20–50 EVs to reach the consensus Ground Truth^18^. In total, the manual annotations took 32 hours for all three datasets (6 hours for Dataset 1, 9 hours for Dataset 2 and 17 hours for Dataset 3).

## Methods

**Figure 1:**
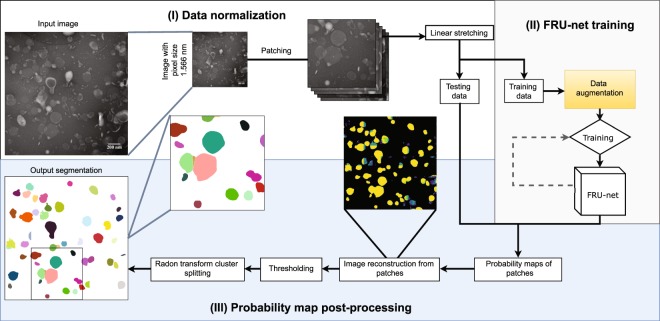
Diagram of the workflow dedicated to the detection and segmentation of small extracellular vesicles in transmission electron microscopy images: (**I**) Data normalization: Every input image is rescaled to a pixel size of 1.56 nm and split into patches of the same size (400 × 400 pixels); (**II**) Fully Residual U-Net (FRU-Net) training; Every patch belonging to the images in the training set is transformed to augment the training data size and FRU-Net is trained; (**III**) Probability map post-processing: All the patches in the test set are processed with the trained FRU-Net. A probability map is obtained after reconstruction of the patches. A binary mask is obtained by thresholding the probability map. Finally, the mask is processed using the Radon transform to facilitate the separation of the clustered vesicle in their individual components.

Here, the proposed method for sEV detection and segmentation in TEM images is explained in detail. A representative diagram of the main steps listed below is shown in Fig. 1:

1. Normalization of TEM images to have the same pixel size and intensity value range.
2. Training of the FRU-Net with image patches and data augmentation.
3. Reconstruction and binarization of the probability maps obtained from the processed patches.
4. Computation of the Radon transform on the binary mask to split clustered vesicles.

### Data normalization

Input image data is normalized to help the algorithm in learning the patterns characterising the information of interest, such as the size of the objects to segment. In the case of sEVs, their rounded shape and fixed size range (~30–200 nm) are the most determinant characteristics and therefore, a common pixel size (1.56 nm) is set in all the images by nearest-neighbor resizing. Hence, the network correctly learns to discard those objects that are too small or large for being a sEV. We divide the resized images into overlapping patches of 400 × 400 pixels (see Supplementary Fig. S3), thus fixing the input size of the FRU-Net. Contrast enhancement and normalization of the individual patch intensity values is obtained by the linear stretching of the intensity histogram to the $[0,1]$ interval.

### Fully residual U-Net

**Figure 2:**
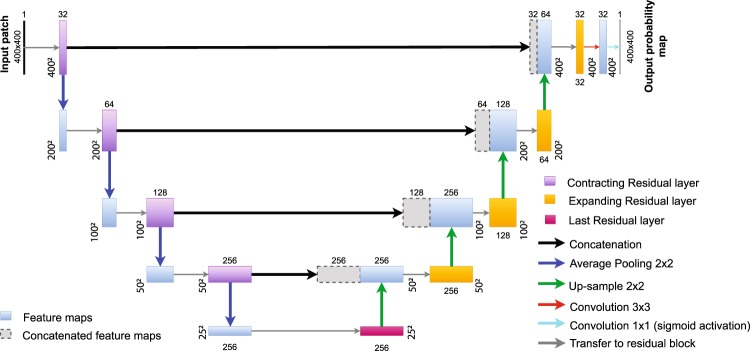
Fully Residual U-Net architecture. Input size is written on the side of each box. The number of feature maps in each convolutional layer is written on the top of each box. Blue and gray boxes represent sets of feature maps. Residual layers (purple, pink and orange boxes) form a set of a convolutional layers with their residual extension, shown in detail in Supplementary Fig. S2. The flow for each new input image patch starts in the black box (top left) and finishes in the white box (top right).

The proposed Fully Residual U-Net (FRU-Net) is inspired by the Fully Residual Convolutional Neural Network (FR-CNN)^42^ and the U-Net^38,39^. Namely, the final architecture is built over a U-shaped deep fully connected CNN. Then, residual layers are added at each level in both the contracting and expanding paths, see Fig. 2. The FRU-Net outputs a probability map in which each value expresses the probability for a pixel to belong to a sEV.

Because the vesicle appearance, in terms of image quality, is quite similar across all the images analyzed, we decided to simplify the original U-Net architecture. Namely, we have decreased by half the number of feature maps and removed the last convolutional filter from each of the levels, see Fig. 2 and Supplementary Fig. S2. Moreover, rectified Linear Units (ReLUs) (commonly used for speeding up the learning process^43^) have been replaced by Exponential Linear Units (ELUs) activation functions ($f(x)=x\,{\rm{for}}\,x > 0$; $f(x)=\alpha ({e}^{x}-1)\,{\rm{for}}\,x\le 0$), to achieve better generalization performance and thus, improve learning^44^.

The residual layers in the FRU-Net are of the form: ELU-Convolution-DropOut-ELU-Convolution. The residual connection in all residual layers is the sum operation without any scaling of the residual output as shown in Supplementary Fig. S2. The general structure of the network is:

- **Contracting path**. Each level in the contracting path is composed of a 3 × 3 convolutional filter set and its residual layer. At the end of each level, the output images are down-sampled by an average-pooling of size 2 × 2, as recommended by^42^. Hence, the patch input size decreases by a factor of two. Small extracellular vesicles in 2D TEM images have a smooth circular shape, thus such down-sampling leads to a better generalization of feature propagation. After down-sampling, the feature map size in each level is increased by a factor of two (i.e., it goes from 32 to 256 in the contracting path).
- **Connection step**. The last residual layer of the FRU-Net differs from the ones in the contracting path. Namely, the feature map size is not doubled at the residual layer input.
- **Expanding path**. The feature maps of the expanding path are built by concatenating the features at the same level in the contracting path with the up-sampled version of the features found at the previous level in the expanding path. Residual blocks are not symmetric with respect to those in the contracting path. Namely, residual layers take their input directly from the concatenation of the high-level and the expanding features. Consequently, in the residual block, an independent 1 × 1 convolutional layer that reduces the number of feature maps and adds the information from the contracting path without losing tiny details is included. Likewise, the first 3 × 3 convolution in the residual layer diminishes the feature map size to make the residual connection possible (see Supplementary Fig. 2).

The last step of the FRU-Net includes a 3 × 3 convolution to refine the feature vectors obtained as the output of the expanding path. Finally, a 1 × 1 convolution with a *sigmoid* activation function ($\sigma (x)=1/\mathrm{(1}+{{\rm{e}}}^{-x})$) determines the probability of the pixel the feature vector corresponds to, to belong to the sEV class (see Fig. 2). We use zero padding in every convolutional layer of the FRU-Net, so the output probability map has the same size as the input patch.

#### Training the FRU-Net

All residual layers are set with 10% of dropout and the *α* parameter of the ELU activation function to 1. As the goal is to obtain a binary classification, the network is trained with *binary cross entropy* as the loss function and *Adam* as optimizer function. The FRU-Net is iteratively trained with both, real and augmented data^45^. Further details about the data augmentation procedure are given in detail in the Supplementary Material.

To evaluate the performance of the FRU-Net during training, 10% of the real patches are saved aside (before data augmentation) and constitute the validation set.

### Post-processing

Our method outputs a labeled mask of the same size as the original image. To this end, the rescaled input image is split into patches that are processed by the FRU-Net and reconstructed afterwards to obtain a final probability map. See Supplementary Material for further details.

Pixels in the given map with a probability value below a fixed threshold $\tau$ are set to zero (background), whereas the others are set to one (sEV). Objects touching the image borders are removed. To smooth object contours, a morphological closing with a ball of radius two is applied. The holes are filled and the objects with an area smaller than *π*(15)^2^ nm^2^ are removed.

Next, the ability of the Radon transform to detect circle-shaped objects is exploited to split clustered sEV^46–49^. As a general idea, the numerical value of each specific pixel in the sinogram represents the projection of all the pixels in the image along a line determined by its slope and position. Therefore, the sum of all the pixels along the line that separates two sEVs is a local minimum in the sinogram. More precisely, the contact area between two touching rounded objects is represented by a hole in the sinogram, see Supplementary Fig. S4. Therefore, we test whether such a local minimum exists in the sinogram of each connected component in the mask. Further details about the implementation are given in the Supplementary Material. The final labelled mask is rescaled to the original size using the nearest neighborhood technique. The boundary of each detected vesicle is locally smoothed by applying first a Gaussian filter with $\sigma =3$, and then, thresholding the blurred region with a threshold, *t*, slightly lower than 0.5 to keep the original object size ($t=0.4$).

## Results

### Evaluation framework

The results are quantitatively evaluated both from a computer science point of view by computing detection and segmentation accuracy and also by testing their biological relevance in terms of vesicle diameter and roundness estimation.

Following the evaluation protocol established within the Cell Tracking Challenge^36^, detection accuracy (DET) is understood as how accurately individual vesicles have been identified. DET estimation is based on a comparison of the nodes of acyclic oriented graphs representing sEVs in both the Ground Truth and the labeled masks produced by the algorithm under assessment. Numerically, DET is defined as a normalised Acyclic Oriented Graphs Matching (AOGM_D_) measure for detection^50^:

1 $${\rm{DET}}=1-\frac{{\rm{\min }}({{\rm{AOGM}}}_{{\rm{D}}},{{\rm{AOGM}}}_{{\rm{0}}})}{{{\rm{AOGM}}}_{{\rm{0}}}}$$

where AOGM_D_ is the cost of transforming a set of nodes provided by the algorithm into the set of Ground Truth nodes, and AOGM_0_ is the cost of creating the set of Ground Truth nodes from scratch (i.e., it is AOGM_D_ for empty results). The minimum operator in the numerator prevents from having a final negative value when it is cheaper to create the Ground Truth set of nodes from scratch than to transform the computed set of nodes into the Ground Truth one. The normalization ensures that DET always falls in the $[0,1]$ interval, with higher values corresponding to better detection performance.

Segmentation accuracy (SEG), understood as how well the segmented regions of individual vesicles match the Ground Truth regions, is based on the Jaccard coefficient^51^. Numerically, the SEG measure is defined as the mean Jaccard coefficient over all Ground Truth sEVs^36^. The SEG measure falls in the $[0,1]$ interval, with higher values corresponding to better segmentation performance. Finally, SEG* corresponds to SEG limited to correctly detected vesicles. Thus, the quality of truly segmented vesicles is assessed.

The possibility of accurately estimate the individual sEV morphology on large samples would have a clear positive impact on sEV related research. In this work, the biological relevance of the results is evaluated by the accuracy of the diameter ($d=2\sqrt{A/\pi }$) and roundness ($r=4\pi A/{P}^{2}$) estimation per sEV. *A* and *P* are the area and the perimeter of the object, with 4*π* being a normalization factor. The errors in the estimation of the diameter and roundness of the correctly detected objects (true positives), *δ*_*d*_ and *δ*_*r*_, respectively, are measured as follows:

2 $${\delta }_{d}=1-\frac{{\rm{\min }}({d}_{Si},{d}_{GTi})}{{\rm{\max }}({d}_{Si},{d}_{GTi})},\,{\delta }_{r}=1-\frac{{\rm{\min }}({r}_{Si},{r}_{GTi})}{{\rm{\max }}({r}_{Si},{r}_{GTi})}$$

where *d*_*GTi*_ and *r*_*GTi*_ are the diameter and roundness of each correctly detected sEV in the Ground Truth, and *d*_*Si*_ and *r*_*Si*_ are the diameter and roundness of its segmentation. Through this formulation, a perfect match would have a null error, whereas a poor match would result in an error close to one. Finally, the average error measured over all correctly detected objects is given.

Additionally, the obtained distributions of sEVs diameters and roundness indices are compared with the Ground Truth values by means of the Wilcoxon Rank Sum test^52^. The null hypothesis in this test states that Ground Truth’s and empirical distributions are the same. The *p*-*values* obtained provide the probability of this being true. Therefore, we can reject this statement with a 95% chance of statistical significance whenever this probability (*p*-*value*) is below 0.05. See Supplementary Fig. S5. To deal with the large number of segmented vesicles and the presence of bias and outliers, the statistical *p*-*value* was estimated by Monte Carlo cross-validation^53^. On each fold of the cross-validation, 30 values are randomly chosen from each set (i.e., the Ground Truth and the set of segmented sEVs). For sample sizes smaller than 30, 2/3 of the sample are chosen in each fold of the cross-validation. The number of folds is given by:

3 $${\rm{folds}}={\rm{floor}}(\frac{10\ast {\rm{sample}}\,{\rm{size}}}{{\rm{fold}}\,{\rm{size}}}),$$

where floor(*x*) is the function that outputs the largest integer value being smaller or equal to the real value *x*.

The averaged *p*-*value* over all the folds is given as result. The *p*-*values* are computed both over all segmented objects and correctly detected objects.

### Quantitative results

To compare the performance of the FRU-Net, the U-Net and TEM ExosomeAnalyzer were also evaluated. Further details about both methods and their settings is included in the Supplementary Material. Additionally, to compare the performance of the deep-learning methods over the entire set of images, three different FRU-Net and U-Net models were trained. We denoted FRU*i* and U*i*, $i=1,2,3,$ the FRU-Net and the U-Net models respectively, tested with Dataset *i*. All the images of the other two datasets that did not belong to the test dataset were included in the training phase. In this way, every image was utilized at least once for both network training and testing. TEM ExosomeAnalyzer was also evaluated over all datasets. Supplementary Table S1 summarizes the distribution of the datasets used for training and testing individual deep-learning models.

Training data together with the augmented data resulted in 3415 patches to train FRU1 and U1, and 2925 patches for the rest of the models. The learning rate was set to 0.0001. Every FRU-Net model was trained during 100 epochs with a batch size of 10. The value of the threshold $\tau$ to binarize the output probability map was calculated as the one which lead to the best detection accuracy: $\tau =0.6$ for FRU1 and U1, and $\tau =0.5$ for the rest.

**Figure 3:**
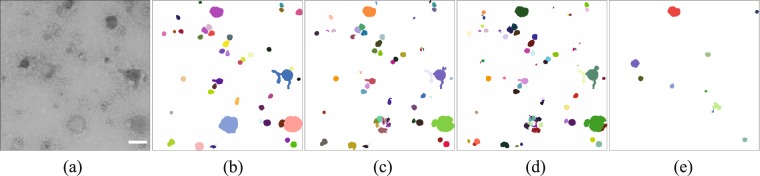
Qualitative evaluation of the segmentation results produced by the three compared methods over a single image in Dataset 3. From left to the right: (**a**) Original image with the pixel size 1.98 nm; scale bar is 500 nm; (**b**) Ground truth; Results of: (**c**) Fully Residual U-Net; (**d**) U-Net; (**e**) TEM ExosomeAnalyzer.

Deep-learning methods detected more objects than TEM ExosomeAnalyzer, see Fig. 3 and Supplementary Fig. S7. Those objects detected by TEM ExosomeAnalyzer are almost circle-shaped and have a high contrast in the original image. The U-Net detected vesicles partially, which impeded the accurate segmentation of images. Namely, it only succeeded in segmenting accurately the objects in Dataset 3. The FRU-Net is the method with the highest rate of correctly-detected sEVs and these are, in most cases, accurately segmented. See Supplementary Material for further details about the SEG* measure and how to interpret it. Large vesicles are among the most challenging objects to segment. For example, none of the methods managed to segment the large blue sEV in Fig. 3(a). The staining is sometimes restricted to the boundaries of the vesicle and the object is then visualized with low contrast. Moreover, if the vesicle is especially large, the network may not be able to detect it. Hence, CNNs may detect part of the boundaries of the sEV but not entirely and therefore, the post-processing may result in a cluster of objects, as shown in Fig. 3(b,c).

Table 1 summarizes the results. In terms of computer-science-oriented evaluation measures, FRU-Net outperformed the methods compared. As for the segmentation accuracy, whereas SEG values varied between 0.62 and 0.74, its SEG* value exceeded 0.84. In the detection task, DET for FRU-Net (between 0.65 and 0.81) was considerably higher than the one achieved with the remaining methods. TEM ExosomeAnalyzer results were more accurate than those of the U-Net when the images contained a low vesicle density (Dataset 1, SEG = 0.52 vs. 0.32; DET = 0.69 vs. 0.32) or lacked artifacts (Dataset 2, SEG = 0.41 vs. 0.33; DET = 0.47 vs. 0.17). By contrast, for images with a high density of vesicles and with artifacts, TEM ExosomeAnalyzer was outperformed by the U-Net (Dataset 3, SEG = 0.19 vs. 0.69; DET = 0.17 vs. 0.77).

**Table 1:** Summary of the performance of each compared method (Fully Residual U-Net (FRU-Net), U-Net and TEM ExosomeAnalyzer (EA)) on different datasets (D1, D2 and D3).

Method	All objects				Correctly detected vesicles						
Dataset 1 (65)	SEG	DET	*p*_*d*_	*p*_*r*_	SEG*	*δ*_*d*_	*δ*_*r*_	TP	FP	*p*_*d*_	*p*_*r*_
FRU-Net	**0.62**	**0.75**	**0.530**	**0.184**	**0.86**	**0.08**	**0.04**	**47**	4	**0.604**	**0.463**
U-Net	*0.32*	*0.32*	0.032**	1.6e^−05^**	0.83	0.09	0.16	*25*	*74*	*0.301*	1e^−04^**
EA	0.52	0.69	0.166	3e^−11^**	*0.76*	*0.15*	*0.26*	45	**3**	0.375	3e^−11^**
**Dataset 2 (346)**											
FRU-Net	**0.62**	**0.65**	**0.312**	**0.468**	**0.84**	**0.29**	**0.08**	**241**	254	**0.507**	*0.103*
U-Net	*0.33*	*0.17*	2.3e^−05^**	*0.239*	*0.71*	*0.30*	*0.14*	*141*	*645*	0.067*	**0.175**
EA	0.41	0.47	0.138	0.379	0.83	**0.29**	0.09	167	**90**	0.454	0.161
**Dataset 3 (688)**											
FRU-Net	**0.74**	**0.81**	**0.490**	0.488	**0.88**	**0.08**	**0.07**	**578**	**159**	**0.488**	*0.348*
U-Net	0.69	0.77	0.434	**0.500**	0.83	0.11	*0.08*	565	257	0.475	0.440
EA	*0.19*	*0.17*	0.0005**	*0.198*	*0.77*	*0.17*	**0.07**	*149*	*335*	*0.334*	**0.493**

SEG: Jaccard coefficient over all Ground Truth sEVs. DET: Acyclic Oriented Graphs Matching measure. SEG*: Jaccard coefficient for the correctly-detected sEV. *δ*_*d*_: diameter error. *δ*_*r*_: roundness error. *p*_*d*_: Wilcoxon Rank Sum test’s mean *p*-*value* for diameters after the *k*-fold cross-validation. *p*_*r*_: Wilcoxon rank sum test’s mean *p*-*value* for roundness after the *k*-fold cross-validation. (*) 95% and (**) 99% of statistical significance. TP: True positives. FP: False Positives. In bold, the best performance and in italics, the worst, per dataset.

FRU-Net also achieved the best results by means of biologically relevant evaluation measures. sEV diameter and roundness were correctly estimated and consequently, the diameter and roundness errors were low (FRU1: ${\delta }_{d}=0.08$, ${\delta }_{r}=0.04$; FRU2: ${\delta }_{d}=0.29$, ${\delta }_{r}=0.08$; FRU3: ${\delta }_{d}=0.08$, ${\delta }_{r}=0.07$). Supplementary Fig. S5 shows the diameter and roundness distributions of segmented sEVs.

The distribution of sEV diameters extracted by the U-Net was only well approximated in Dataset 3 (${p}_{d} > 0.05$). With the exception of Dataset 1, the roundness distribution was always well estimated (${p}_{r} > 0.05$). A visual example of these results is given in Fig. 3 and Supplementary Fig. S7.

TEM ExosomeAnalyzer on Dataset 1 (EA1) and Dataset 2 (EA2) and FRU3 achieved the lowest False Positive ratio (FP/(FP + TP)) (EA1: 0.063; EA2: 0.350 and FRU3: 0.216). FRU1 resulted in similar ratios as EA1 (FRU1: 0.078) whereas those corresponding to the rest of the models were considerably worse. Any morphological measure computed from an automatic image processing method, provides data about true and false detections. Therefore, the lower the false positive ratio is, the less biased these morphological values will be. See Supplementary Fig. S6 for a visual comparison of false positives ratios achieved by individual methods.

The experimental evaluation of FRU-Net and U-Net was conducted on a Intel Core i7-6700, 3.4 GHz × 8 workstation and implemented in Python using the Keras library [https://keras.io] with a Titan X (Pascal) GPU 12 GB. Both methods took just 2.1 seconds to segment one entire image of 2048 × 2048 pixels, which means that only 3.5 minutes are required to segment 100 images. Training of the CNN models took, on average, 17 hours (305 sec/epoch) for FRU1, 15 hours (264 sec/epoch) for FRU2 and FRU3, 11 hours (202 sec/epoch) for U1, and 10 hours (174 sec/epoch) for U2 and U3. TEM ExosomeAnalyzer execution time depended on the number of candidate objects detected. On average, it took 8.4 minutes to analyze one single image. A further evaluation of execution and curation time is given in the Supplementary Material.

## Discussion

The answer to novel biological questions about cellular processes goes hand in hand with the new technological developments that enable robust extraction of relevant and high-quality information. In this work, we aimed to transmit this idea to the reader. Specifically, we have presented a robust, deep-learning based approach for the fully automatic quantitative analysis of sEV morphology in TEM images.

First, a large and heterogeneous TEM image set of sEVs together with its corresponding annotations was obtained. The current set could serve as a benchmark for the evaluation of novel segmentation methods and the researchers can find here a source of data to train machine learning techniques. Second, the FRU-Net was trained with different combinations of available data for a complete evaluation of the current work. Its results were compared with those produced by two state-of-the-art methods: TEM ExosomeAnalyzer and the U-Net convolutional neural network. Finally, the quantitative evaluation was carried out from two different perspectives: the classical computer science measures and those oriented toward the biological relevance of the results.

When comparing the U-Net and the FRU-Net, the main difference is that the latter discarded better the debris than the former, see Fig. 3, Supplementary Fig. S7 and Table 1. In other words, the FRU-Net architecture has a higher ability to generalize. This is a consequence of including the residual layers and dropout operation, as they help to reject outliers (non circle-shaped vesicles and artifacts) and hence, avoid sample bias^40^. The U-Net only provided similarly good results to the FRU-Net when Dataset 1 and 2 were used for training. This pair of datasets was the most homogeneous possible training set and consequently, the number of outliers and artifacts was reduced. Furthermore, deep-learning models with the lowest accuracy measures were those (FRU2 and U2) trained with the most heterogeneous image set, which reflects the relevance of building a proper training set. In the case of FRU1 and U1, the reasons for a worse accuracy than that of FRU3 and U3 are twofold: (1) the total number of vesicles is much lower in Dataset 1 than in Dataset 3. Therefore, a single error weights more than what it does in Dataset 3; (2) Even if the training included homogeneous images (Dataset 2), Dataset 3 contained a much higher amount of heterogeneous vesicles, so the training set was probably too biased to allow the CNN to learn to generalize. Yet, the inclusion of some noisy images in the training is always important. To support this statement, we trained our FRU-Net using only the homogeneous Dataset 2 as a training set. The results are shown in Supplementary Table S1. When evaluated in Dataset 1, SEG and DET decreased from 0.62 and 0.75 to 0.556 and 0.590, respectively. The deterioration in the estimation is more pronounced when evaluating the model on Dataset 3: SEG and DET were approximately halved from 0.74 and 0.81 to 0.324 and 0.408, respectively. Namely, the performance is worse than that achieved when including the heterogeneity of Dataset 1 or Dataset 3 in the training (FRU1 and FRU3). As a conclusion, we strongly recommend the reader to focus on the accuracy obtained by FRU3. We believe that this model is the most suitable to analyze new input image data: it has learned to generalize from homogeneous data (Dataset 2) and it has been trained with enough outliers (Dataset 1) as to be able to process the most challenging images (Dataset 3) in an accurate way. In addition to accuracy and robustness, an important factor required is time efficiency^22^. Once they are trained, both FRU-Net and U-Net models are able to automatically process a large amount of images in just few minutes, providing a powerful tool for biomedical research.

TEM ExosomeAnalyzer demonstrated to be a powerful method when working with a low density of vesicles and homogeneous data (Dataset 1 and Dataset 2). When tested on a more heterogeneous scenario (Dataset 3), it is recommended to cure the output. The software has a semi-automatic mode that is well suited for this task and allowed to produce biologically relevant results when we used in^18^. It must be highlighted that TEM ExosomeAnalyzer is an unsupervised method. Therefore, new data can be directly processed by the algorithm without requiring model training or image annotation. Furthermore, it is a standalone and ready-to-use software for computer science non-specialists, unlike advance deep-learning models. It consists of a user-friendly interface that even allows the curation of results, which provides an added value for the community.

In^18^, the information given by TEM images and other characterization techniques (NTA, TRPS and cryo-EM) is obtained for the first time. In particular, it is shown that the measurements from TEM images are equivalent to those provided by NTA, TRPS and cryo-EM, when segmented accurately. Hence, in the present work, to prove the usefulness of the proposed method, sEV diameter and roundness distributions were evaluated as a measure of the biological relevance of the results. In particular, the error in the estimation of the sEV diameter and roundness, *δ*_*d*_ and *δ*_*r*_, were computed. Then, we tested whether the distribution of the diameter *d* and the roundness *r* provided was the same as that in the Ground Truth. Supplementary Fig. S5 and Table 1 show that the diameter distribution of the sEVs in our results can be considered the same as the one in the Ground Truth, and therefore, it can be assumed that our measurements are equivalent to those obtained using traditional approaches.

To sum up, we have presented a robust tool for sEV detection and segmentation in TEM images. After a suitable training based on very few images, the learned model has shown the ability to accurately process highly heterogeneous images. More importantly, the information extracted from the final instance segmentation masks have provide a close estimation of sEV morphology. Moreover, individual vesicle size and shape estimates have no bias when compared to that provided by the classical nanoscale characterization techniques as no prior assumption or calibration is needed. The integration of this tool into the characterization pipelines could help biomedical researchers in defining of sEV sub-populations, which could contribute to deciphering the role of sEVs in patho-physiological processes.

## Supplementary information

Supplementary Material

## Data Availability

The web page: https://cbia.fi.muni.cz/research/segmentation/fru-net provides free access to the datasets, along with the Ground Truth and a ready-to-use Python code for image processing.
