# Supplementary material for: Deep-Learning-Based Segmentation of Small Extracellular Vesicles in Transmission Electron Microscopy Images
Source: Sci Rep. 2019 Sep 13;9:13211. doi: 10.1038/s41598-019-49431-3 (PMC6744556; doi:10.1038/s41598-019-49431-3)
Supplement: Supplementary file 1 — Supplementary Material [file 41598_2019_49431_MOESM1_ESM.pdf]

Deep-Learning-Based Segmentation of Small Extracellular Vesicles in Transmission Electron Microscopy Images - Supplementary Material

Estibaliz Gómez-de-Mariscal[1,2], Martin Maška[3], Anna Kotrbová[4], Vendula Pospíchalová[4], Pavel Matula[3], and Arrate Muñoz-Barrutia[1,2,*]

[1]Bioengineering and Aerospace Engineering Department, Universidad Carlos III de Madrid, Leganés, 28911, Spain
[2]Instituto de Investigación Sanitaria Gregorio Marañón, Madrid, 28007, Spain
[3]Centre for Biomedical Image Analysis, Faculty of Informatics, Masaryk University, Brno, 602 00, Czech Republic
[4]Department of Experimental Biology, Faculty of Science, Masaryk University, Brno, 611 37, Czech Republic
[*]Correspondence and requests for materials should be addressed to A.M.B. (email: mamunozb@ing.uc3m.es)

Materials

Sup. Figure S1 shows an example of the images included in each dataset.

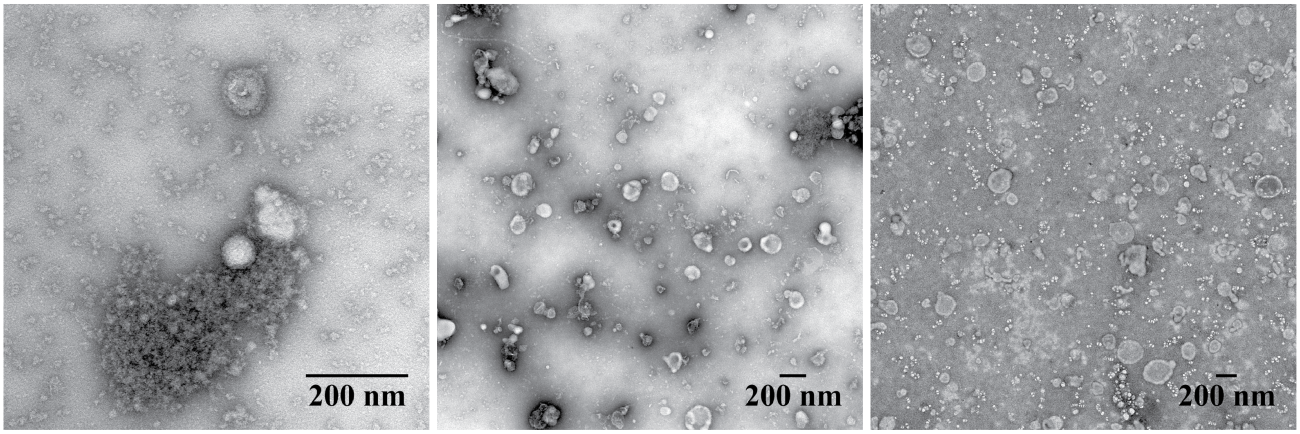

(a) (b) (c)

Sup. Figure S1. Examples of transmission electron microscopy images of small extracellular vesicles belonging to: (a) Dataset 1 (pixel size of 0.40 nm); (b) Dataset 2 (pixel size of1.56 nm) and (c) Dataset 3 (pixel size of 1.98 nm). The background of images (a) and (c) is covered by artifacts with a grainy appearance, whereas (b) is a much cleaner image with a smooth background.

Fully Residual U-Net

Residual layers

The residual extensions in the Fully Residual U-Net (FRU-Net) are of the form ELU-CONVOLUTION-DROPOUT-ELU-CONVOLUTION. However, three different residual layers are implemented being used in the contracting path (Sup. Figure S2(a)), the connection step (Sup. Figure S2(b)) and the expanding path (Sup. Figure S2(c)) of the network.

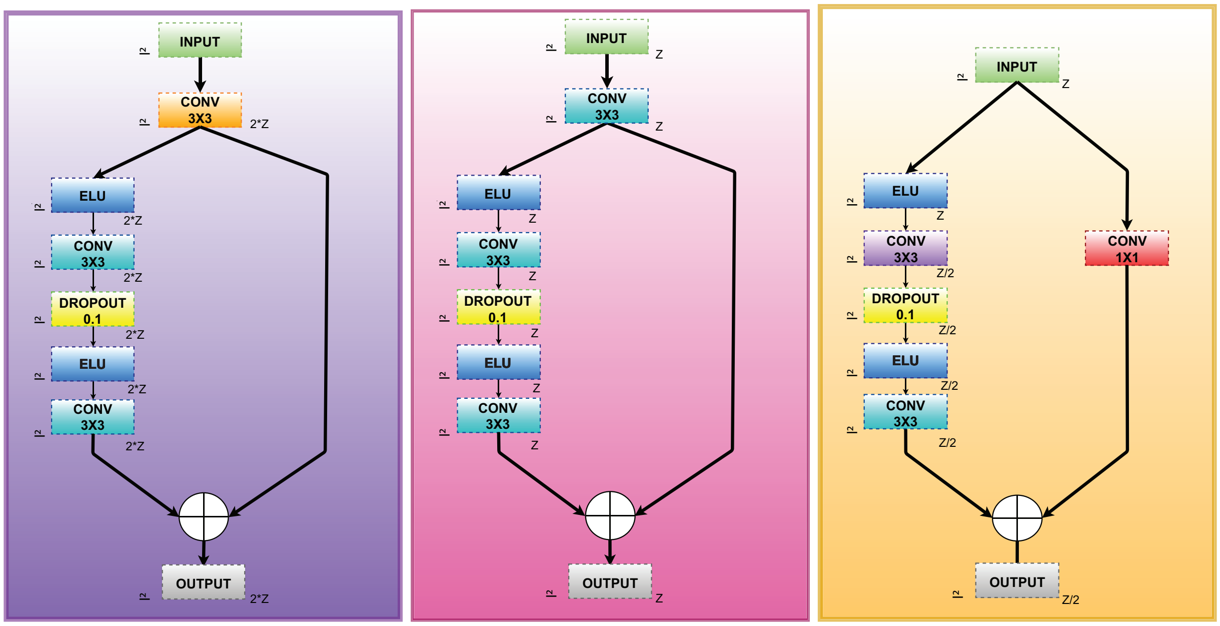

(a) (b) (c)

Sup. Figure S2. Illustration of the residual layers composing the Fully Residual U-Net architecture: (a) Contracting residual layer; (b) Last residual layer in the contracting path where the number of feature maps is not doubled; (c) Expanding residual layer where the feature maps are reduced in both branches of the block: in the residual branch, feature maps are reduced by half in the first 3×3 convolutional layer, and in the other branch, channels are also reduced by half, but with a 1×1 convolutional layer. ELU: Exponential Linear Unit activation function. DROPOUT: Dropout layer. CONV: Set of convolutions.

Data augmentation

The FRU-Net is iteratively trained with both, real and augmented data[1]. New data is obtained as a result of random geometrical transformations of patches. The transformation parameters are set accordingly to the size range of sEVs: rotations (angle range $[\pm 30°]$), width and height shifts (maximum shift of $10\,\%$), shear (shear angle range of $[\pm 0.05°]$), zooming (zoom range of $[0.8, 1.2]$) and horizontal flips. Each image in the final augmented data is the result of a random combination of all the described transformations. Transformations are iteratively applied to the patches 200 times in batches of size 10.

FRU-Net input size is set to 400×400 pixels, so patches of 500×500 pixels are first extracted and after data augmentation, their borders are cropped to the desired size and to avoid the inclusion of spurious objects in the training (see Sup. Figure S3). The procedure to obtain the patches is as follows: If the re-sized image size is smaller than 500×500 pixels, image borders are augmented by mirroring until the desired size is reached; Otherwise, images are split into patches of 500×500 pixels with 125 pixels of overlap from each side. See Sup. Figure S3 for further details.

Reconstruction of probability maps

Through the proposed method, every image is rescaled and split into patches of 400×400 pixels in size with an overlap of 125 pixels on each side, see Sup. Figure S3. Each of those patches are the inputs of the presented Fully Residual U-Net (FRU-Net) and the output is a probability map of the same size. The reconstruction of the rescaled probability map is obtained by taking into account the overlap between the neighboring patches. Each pixel in the patch belongs to a specific location in the rescaled image. If

that pixel does not appear in any other patch, its value is assigned to the specific location in the rescaled probability map. Otherwise, the average over all values of the pixels that belong to the specific location in the rescaled image is taken there. See Sup. Figure S3.

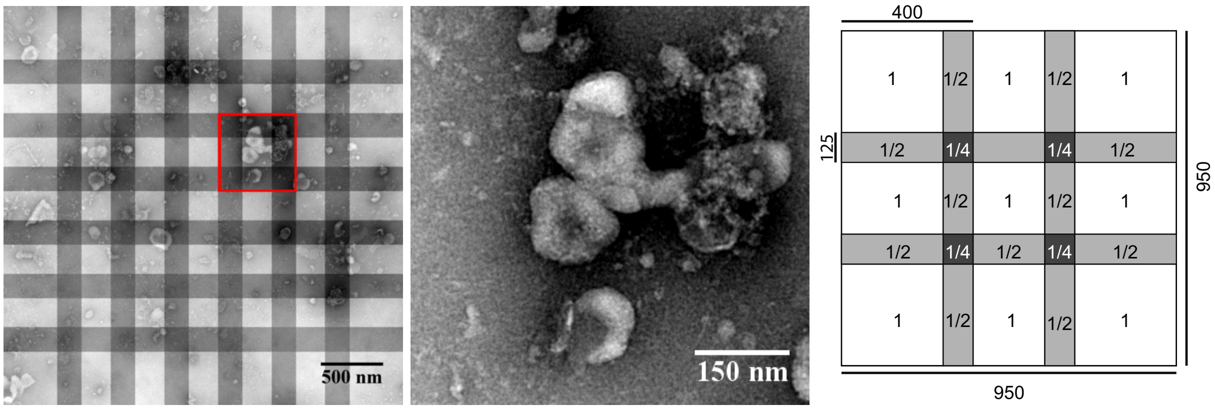

(a) (b) (c)

Sup. Figure S3. Graphical example of the patch splitting procedure: (a) Overlap of the rescaled image and a template containing all patch positions. The red square represents one patch. The gray borders represent the overlap between neighboring patches. (b) Zoom of the patch delimited by the red box in (a). (c) Numerical representation of a template for an image of size 950×950 pixels and patches of 400×400 pixels. The numbers inside the template correspond to the weights assigned to the pixel value that lie within specific regions when the rescaled probability map is being reconstructed.

Probability map post-processing: Cluster splitting

The line that splits two touching rounded objects is represented by a hole in their sinogram, see Sup. Figure S4. Therefore, each connected component (CC_i) in the masks is post-processed as follows:

I Obtain the sinogram (S_i) using the Radon transform of the bounding box of the connected components, CC_i.

II Enhance the contrast of S_i by applying white and black top-hat transforms: $CE(S_i) = S_i + WT(S_i) - BT(S_i)$, where WT and BT are the white and black top-hat filters, respectively, configured with a sufficiently large kernel of disk shape (a circle of 10 pixels in radius).

III Get a binary mask b_i by applying Otsu thresholding[2].

IV Obtain the sinogram's local minimum m_{ij} within each of the holes of the binary mask b_i.

V Reconstruct the line represented by the local minimum m_{ij} and intersect it with the connected component CC_i.

VI Undo the splits that result in too small over-segmentations, taking into account the size range of sEVs.

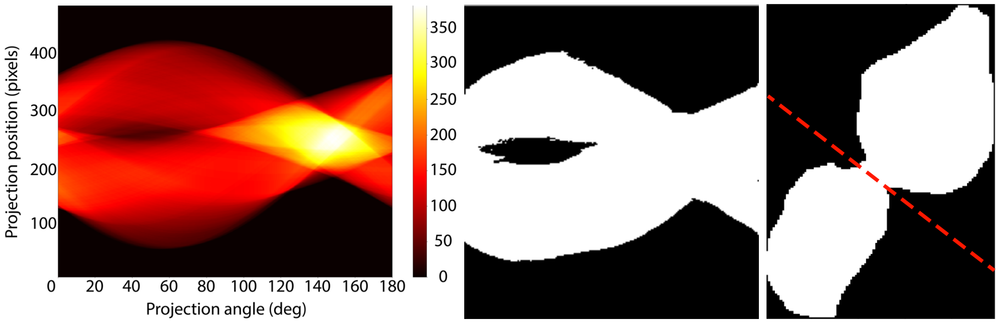

(a) (b) (c)

Sup. Figure S4. Illustration of the vesicle cluster splitting procedure based on the Radon transform: (a) Sinogram of the vesicle cluster in (c) before separation. (b) Mask of (a) with a hole in the area of the local minimum representing the red line that splits the two small extracellular vesicles in (c). (c) Result of the minimum reconstruction over the vesicle cluster, which coincides with the red line splitting it into two vesicles.

Reference methods

In TEM ExosomeAnalyzer[3], TEM images are preprocessed with an edge-enhancing diffusion filter[4] for which the contrast parameter λ needs to be specified (default: 0.005). Then, a gradient magnitude image is calculated and a gradual edge growing is applied to obtain those enclosed objects that represent the needed seeds for a morphological watershed processing. Candidate seeds are filtered out depending on their shape by a morphological opening with a disc structuring element, as sEVs are considered to be of circle shape. The size of the disk, α, is a fraction of the expected sEV size (default: 0.15).

The U-Net[5,6] is a fully connected CNN that consists of contracting and expanding paths. Its architecture is arranged with the same combination of parameters as the FRU-Net to facilitate the comparison between the output of both approaches. Therefore, the contracting path in the U-Net starts with a convolutional layer of 32 channels. Loss and optimizer functions are the same as for the FRU-Net. The validation dataset (i.e. 10% of the real patches before data augmentation is computed) is used to reduce the learning rate and optimize U-Net's performance in the next way: (1) While training the network, the algorithm stores the minimum value of the loss function in the validation dataset, *min-loss*; (2) If the loss value during the next K epochs decreases, *min-loss* is updated; if it is not the case, the learning rate is reduced.

The output probability maps of the U-Net are post-processed following the same steps as for FRU-Net outputs: the reconstructed probability maps are thresholded, clusters are split using the Radon transform, resulting labelled mask is rescaled to the original size and finally, detected vesicles' borders are smoothed.

Visual summary of the obtained results

In this section, we illustrate the performance of the FRU-Net and provide a comparison with two state-of-the-art methods.

- Sup. Table S1 provides the data distribution used for training and testing the supervised methods (U-Net and FRU-Net).
- In Sup. Figure S5, a comparison between the morphological parameters obtained after the FRU-Net processing is presented.

- In Sup. Figure S6 an illustration of the detection performance for each method and dataset is shown.
- Sup. Figure S7 and Sup. Table S2 provide qualitative and quantitative information on the distribution of the Jaccard coefficients for the correctly detected sEVs (SEG*).

Methods	Name	Training dataset	Testing dataset
U-Net	U1	Datasets 2 and 3	Dataset 1
U-Net	U2	Datasets 1 and 3	Dataset 2
U-Net	U3	Datasets 1 and 2	Dataset 3
Fully Residual U-Net	FRU1	Datasets 2 and 3	Dataset 1
Fully Residual U-Net	FRU2	Datasets 1 and 3	Dataset 2
Fully Residual U-Net	FRU3	Datasets 1 and 2	Dataset 3
TEM ExosomeAnalyzer	EA	-	Datasets 1, 2 and 3

Sup. Table S1. Distribution of the datasets used for training and testing of the compared methods.

Although the estimated mean values of the vesicle diameters and roundness indices are slightly biased toward the Ground Truth when using the FRU-Net, the estimated distribution is not significantly different as supported by the *p-values* of the Wilcoxon Rank Sum test with the 5% confidence interval. Moreover, when only correctly segmented vesicles are evaluated, it can be seen that both distributions are almost identical. See Sup. Figure S5.

TEM ExosomeAnalyzer is the method with the lowest number of false positives, followed by the FRU-Net and finally, the U-Net, which is the method with the worst performance in this sense. Both deep-learning models get better results when they are trained with Dataset 2 (i.e., FRU2 and U2 are the ones with the higher ratio of false positives), whereas TEM ExosomeAnalyzer performs similarly in Dataset 2 and 3. TEM ExosomeAnalyzer is more sensitive to a higher density of vesicles, whereas the deep-learning models strongly depend on the heterogeneity of the training data. See Sup. Figure S6.

Dataset 1	Mean	Standard dev.	Lower quartile	Median	Upper quartile	Minimum	Maximum
FRU-Net	**0.860**	0.154	**0.871**	**0.906**	**0.931**	0.089	**0.973**
U-Net	0.828	**0.101**	0.776	0.857	0.897	**0.513**	0.967
EA	0.757	0.108	0.655	0.793	0.823	0.510	0.947
Dataset 2							
FRU-Net	**0.838**	0.098	0.804	**0.868**	**0.903**	**0.488**	**0.954**
U-Net	0.714	0.144	0.574	0.736	0.842	0.389	0.942
EA	0.833	0.112	**0.811**	0.864	0.901	0.156	0.945
Dataset 3							
FRU-Net	**0.882**	0.115	**0.878**	**0.917**	**0.946**	**0.084**	**0.984**
U-Net	0.831	0.143	0.806	0.880	0.918	0.033	0.968
EA	0.769	**0.110**	0.694	0.794	0.850	0.395	0.952

Sup. Table S2. Statistics of the Jaccard coefficient distributions of the correctly detected sEVs (SEG*).

As shown in Table 1 in the main manuscript, FRU-Net is the most accurate segmentation method (highest SEG in all the cases). However, when SEG* is analyzed, the difference between all the tested methods is much smaller. In particular, the main difference when comparing FRU-Net with the rest of the methods is that it segments accurately most of the correctly detected sEVs (true positives). The quartiles of SEG* are substantially higher than for the rest of the methods. See Sup. Table S2.

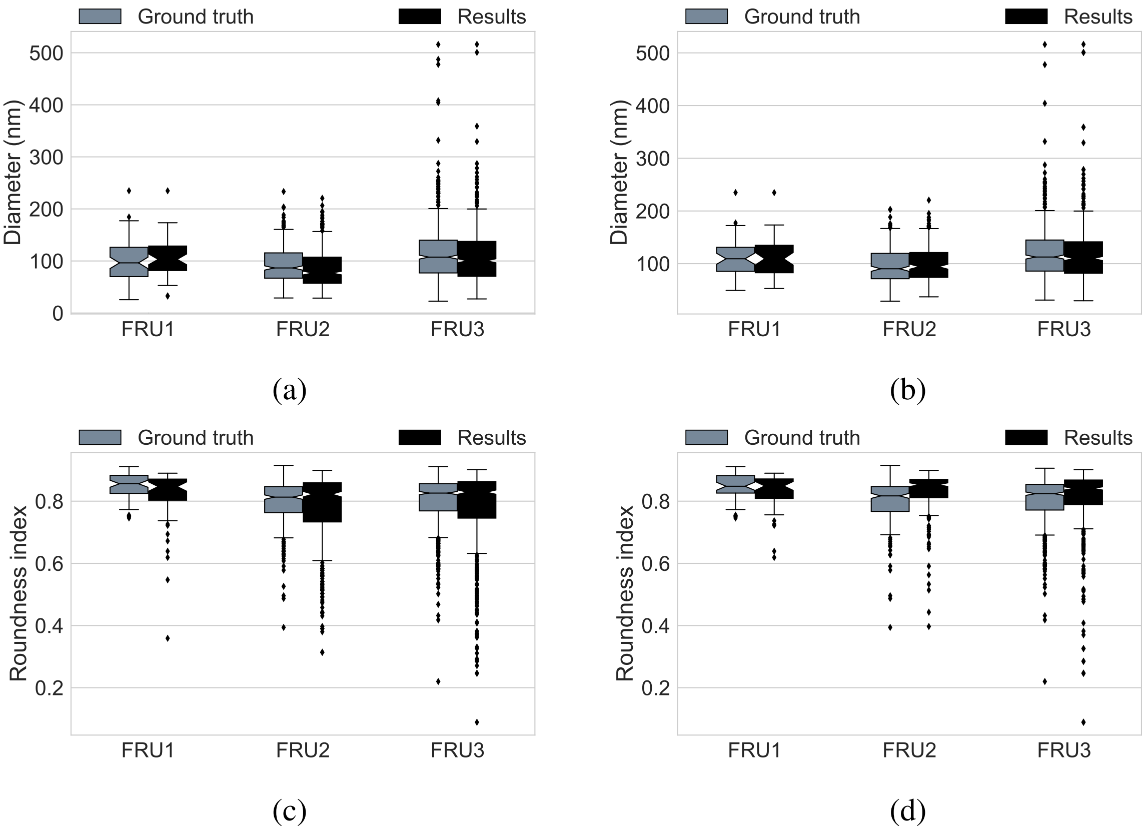

Sup. Figure S5. Boxplot representation of diameter and roundness for all the vesicles in the ground truth (in gray) and in the FRU-Net results (in black). Notches are calculated for the 95% confidence interval of the median and whiskers are limited to one standard deviation over the 25% and 75% quantiles. Diameter for: (a) all detected objects; (b) correctly detected vesicles in the ground truth; Roundness index for: (c) all detected objects and (d) correctly detected vesicles in the ground truth.

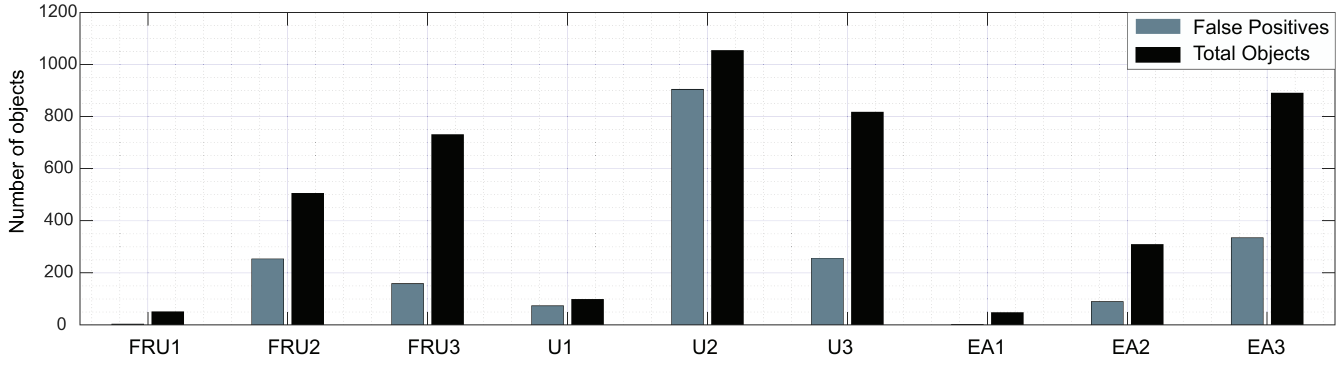

Sup. Figure S6. Graphical representation of false positive detection. In black, the number of objects detected by each method. In gray, the number of false positives.

Evaluation of execution time

This section provides a comparison between the time required to annotate TEM images manually and the time needed first, to train a deep learning model and second, to analyze new images with the trained model. The input parameters needed for the estimation of execution time can be found in Sup. Table S3.

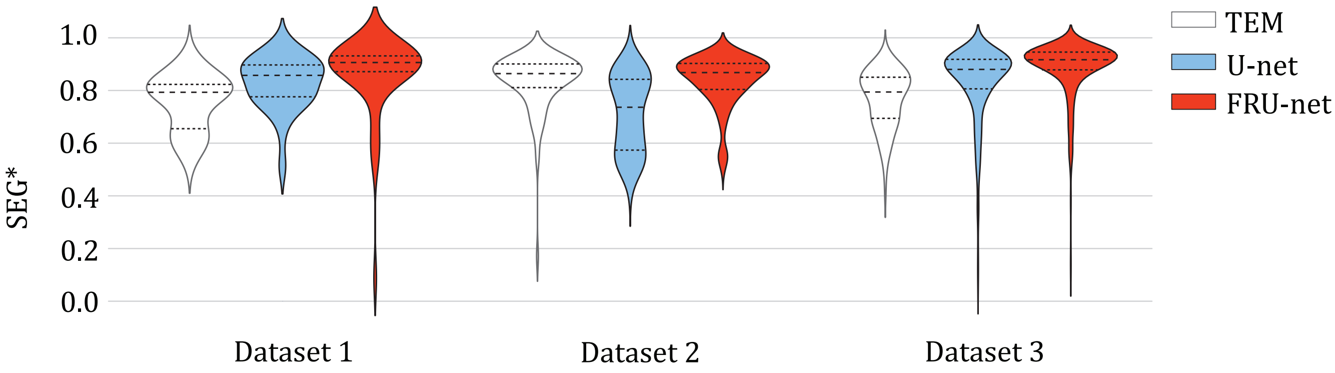

Sup. Figure S7. Violin plots of the Jaccard coefficient for the correctly detected vesicles (SEG*). Dashed lines in the plots represent the three quartiles as detailed in Sup. Table S2.

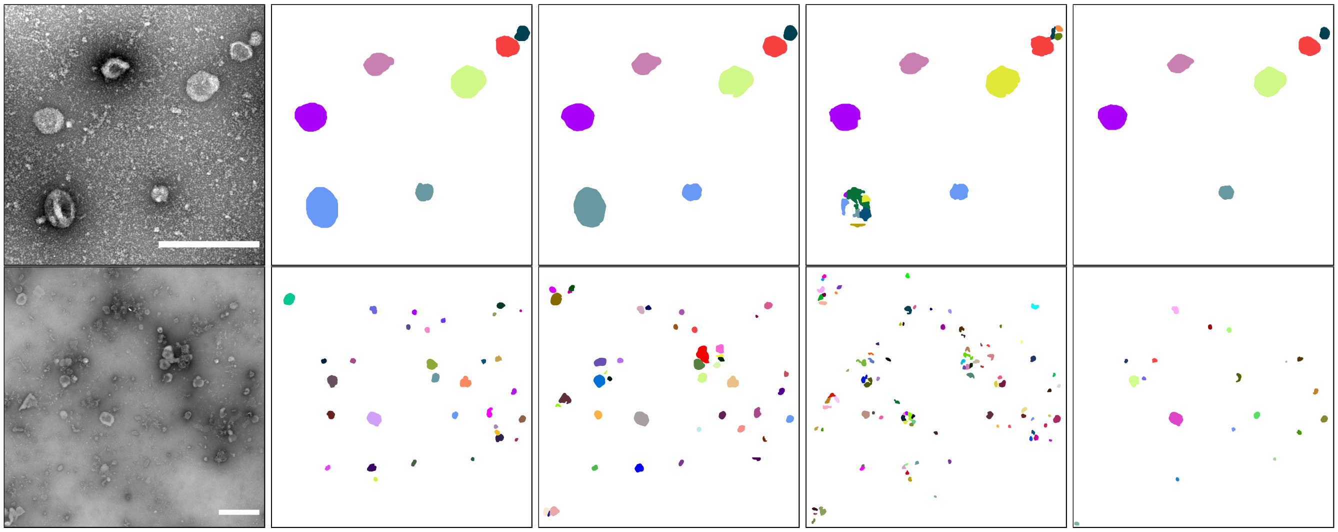

Sup. Figure S8. Qualitative evaluation of the segmentation results produced by the three compared methods over a single image in Datasets 1 (top) and 3 (bottom). (a) Original images with the pixel sizes of 0.41 and 1.56 nm, respectively; scale bars are 500 nm; (b) Ground truth; Results of: (c) Fully Residual U-Net; (d) U-Net; (e) TEM ExosomeAnalyzer.

Assuming that removal of a single false positive object as well as addition of a single detection marker takes 1 s, the average curation time per sEV in the FRU3 results is $\mathrm{NTCF}_M = 23.42$ s (Equation 1) when the curation is performed manually,

$$NTCF_M = \frac{TP_F(1 - \mathrm{SEG}_F^*)NTM + FP_F + FN_F NTM}{N} \quad (1)$$

or $\mathrm{NTCF}_{EA} = 17.63$ s (Equation 2) when the curation is performed semi-automatically using TEM ExosomeAnalyzer,

$$NTCF_{EA} = \frac{TP_F(1 - \mathrm{SEG}_F^*)NTM + FP_F + FN_F NTEA_M}{N} \quad (2)$$

Parameter	Value	Description
T	15 hours	The time required for training the FRU3 model
TF	2.1 s (41.1 s)	The average analysis time of the FRU3 model running on GPU (CPU) per image in Dataset 3
TEA	8.4 min	The average analysis time of TEM ExosomesAnalyzer running on CPU per image in Dataset 3
TM	17 hours	The total time required for manually annotating all images in Dataset 3
N	688	The total number of sEVS in Dataset 3
NTF	0.11 (2.12 s)	Normalized TF per sEV running on CPU (GPU)
NTEA	39.57 s	Normalized TEA per sEV
NTM	88.95 s	Normalized TM per sEV
NTCF_M	23.42 s	Normalized manual curation time per sEV in FRU3 results
NTCF_{EA}	17.63 s	Normalized curation time per sEV in FRU3 results using TEM Exosome Analyzer
NTCEA_M	52.73 s	Normalized manual curation time per sEV in TEM ExosomeAnalyzer results
$(\text{TP}_F, \text{FP}_F, \text{FN}_F, \text{SEG}^*_F)$	$(578, 159, 110, 0.88)$	The number of true positive, false positive, and false negative objects produced by the FRU3 model and the average SEG* score over true positive objects
$(\text{TP}_{EA}, \text{FP}_{EA}, \text{FN}_{EA}, \text{SEG}^*_{EA})$	$(149, 335, 539, 0.77)$	The number of true positive, false positive, and false negative objects produced by TEM ExosomeAnalyzer and the average SEG* score over true positive objects

Sup. Table S3. Definition of input parameters to test the required execution and manual annotation time.

and NTCEA_M = 52.73 s (Equation 3) being the average curation time per sEV in the TEM Exosome-Analyzer results when the curation is performed manually,

$$NTCEA_M = \frac{TP_{EA}(1-\text{SEG}^*_{EA})NTM + FP_{EA} + FN_{EA} + FN_{EA}NTEA + FN_{EA}(1-\text{SEG}^*_{EA}) * NTM}{N} \quad (3)$$

Averaged curation times NTCF_M, NTCF_{EA} and NTCEA_M lead us to the following conclusions:

- Without the need for training a new model, it is always (i.e., for any positive number of sEVs) faster to analyze images using FRU3 (running on both GPU and CPU) and curate the results, either manually or semi-automatically using TEM ExosomeAnalyzer, than manually annotating the images. That is, the Equations 4 and 5 hold:

$$NTM \leq (NTF + NTCF_M) \quad (4)$$
$$NTM \leq (NTF + NTCF_{EA}) \quad (5)$$

- By taking into account 15 hours of training time for FRU3 (on GPU), the analysis of images using FRU3 and subsequent curation of the results is faster than manually annotating the images:

- If more than 825/851 sEVs are analyzed (FRU3 running on GPU/CPU) and the curation is performed manually (solution for x_M in Equation 6):

$$x_M NTM \leq x_M(NTF + NTCF_M) + T \tag{6}$$

- If more than 758/780 sEVs are analyzed (FRU3 running on GPU/CPU) and the curation is performed semi-automatically using TEM ExosomeAnalyzer (solution for x_{EA} in Equation 7):

$$x_{EA} NTM \leq x_{EA}(NTF + NTCF_{EA}) + T \tag{7}$$

Training with Dataset 2

The models trained with a dataset including the images in Dataset 2 were the most accurate ones, as shown in the main manuscript. Simultaneously, this dataset is the only one containing homogeneous images of small extracellular vesicles (sEVs). Therefore, the FRU-Net was trained once more, using only the images from Dataset 2, to evaluate the performance of a model just trained on homogeneous TEM images. The parameters used were the same as those used for the other models. As it can be seen in Sup. Table S4, the accuracy measures are lower for both Datasets 1 and 3 than when we process them with FRU1 and FRU3, respectively. See Table 1 in the main manuscript for comparison.

Method	All objects				Correctly detected objects						
Datasets 1 and 3 (n = 753)	SEG	DET	p_d	p_r	SEG*	δ_d	δ_r	TP	FP	p_d	p_r
FRU-Net	0.345	0.42	0.201	0.416	0.832	0.092	0.067	357	371	0.437	0.461
Dataset 1 (n = 65)	SEG	DET	p_d	p_r	SEG*	δ_d	δ_r	TP	FP	p_d	p_r
FRU-Net	0.556	0.590	0.201	0.416	0.881	0.072	0.039	40	8	0.492	0.729
Dataset 3 (n = 688)	SEG	DET	p_d	p_r	SEG*	δ_d	δ_r	TP	FP	p_d	p_r
FRU-Net	0.324	0.408	0.178	0.422	0.824	0.094	0.071	317	363	0.450	0.420

Sup. Table S4. Summary of the FRU-Net trained with the images from Dataset 2. SEG : Jaccard coefficient over all ground truth sEVs. DET : Acyclic Oriented Graphs Matching measure. SEG* : Jaccard coefficient for the detected vesicles. δ_d: diameter error. δ_r: roundness error. p_d: Wilcoxon Rank Sum test's mean *p-value* for diameters after the *k*-fold cross-validation. p_r: Wilcoxon rank sum test's mean *p-value* for roundness after the *k*-fold cross-validation. (*) 95% and (**) 99% of statistical significance. TP: True positives. FP: False Positives. In blue, the best performance and in red, the worst, for each set of images.

References

1. Dosovitskiy, A., Springenberg, J. T., Riedmiller, M. & Brox, T. Discriminative unsupervised feature learning with convolutional neural networks. In *Adv. Neural Inf. Process. Syst. 27 (NIPS 2014)*, 766–774 (2014).

2. Otsu, N. A threshold selection method from gray-level histograms. *IEEE Transactions On Syst. Man, Cybern.* **9**, 62–66 (1979).

3. Štěpka, K. *et al.* Automatic detection and segmentation of exosomes in transmission electron microscopy. In Hua, G. & Jégou, H. (eds.) *ECCV 2016 Work.*, vol. 9913 of *Lecture Notes in Computer Science*, 318–325, DOI: 10.1007/978-3-319-46604-0_23 (Springer International Publishing, Cham, 2016). 1608.03819.

4. Weickert, J. & Stuttgart, B. G. T. *Anisotropic diffusion in image processing*. Ph.D. thesis.

5. Ronneberger, O., Fischer, P. & Brox, T. U-Net: convolutional networks for biomedical image segmentation. In Springer (ed.) *Int. Conf. Med. image Comput. Comput. Interv.*, 234–241, DOI: 10.1007/978-3-319-24574-4_28 (Springer International Publishing, 2015). 1505.04597.

6. Falk, T. *et al.* U-Net: deep learning for cell counting, detection, and morphometry. *Nat. Methods* **16**, 67–70, DOI: 10.1038/s41592-018-0261-2 (2019).
